# Evaluating Methods to Correct for Population Stratification when Estimating Paternity Indexes

**DOI:** 10.1371/journal.pone.0049832

**Published:** 2012-11-30

**Authors:** Ulises Toscanini, Manuel Garcia-Magariños, Gabriela Berardi, Thore Egeland, Eduardo Raimondi, Antonio Salas

**Affiliations:** 1 Pricai-Fundación Favaloro, Ciudad Autónoma de Buenos Aires, Buenos Aires, Argentina; 2 Unidade de Xenética, Instituto de Medicina Legal, Facultad de Medicina, Universidad de Santiago de Compostela, Galicia, Spain; 3 Norwegian University of Life Sciences, IKBM, Aas, Norway; Kunming Institute of Zoology, Chinese Academy of Sciences, China

## Abstract

The statistical interpretation of the forensic genetic evidence requires the use of allelic frequency estimates in the reference population for the studied markers. Differences in the genetic make up of the populations can be reflected in statistically different allelic frequency distributions. One can easily figure out that collecting such information for any given population is not always possible. Therefore, alternative approaches are needed in these cases in order to compensate for the lack of information. A number of statistics have been proposed to control for population stratification in paternity testing and forensic casework, *Fst* correction being the only one recommended by the forensic community. In this study we aimed to evaluate the performance of *Fst* to correct for population stratification in forensics. By way of simulations, we first tested the dependence of *Fst* on the relative sizes of the sub-populations, and second, we measured the effect of the *Fst* corrections on the Paternity Index (*PI*) values compared to the ones obtained when using the local reference database. The results provide clear-cut evidence that (i) *Fst* values are strongly dependent on the sampling scheme, and therefore, for most situations it would be almost impossible to estimate real values of *Fst*; and (ii) *Fst* corrections might unfairly correct *PI* values for stratification, suggesting the use of local databases whenever possible to estimate the frequencies of genetic profiles and *PI* values.

## Introduction

According to current recommendations [1] the statistical interpretation of the genetic evidence in forensic genetics (i.e. paternity testing, forensic casework) should be based on the calculation of likelihood ratios (*LR*) between the probabilities of two contrasting hypotheses. Usually, hypotheses are formulated as if the evidence comes from a given suspect (e.g. in forensic casework) *versus* the evidence originating from some randomly selected individual from the relevant population. Calculation of the probability for the latter hypothesis requires an estimation of allele frequencies in the reference population, e.g. the population where a crime was committed. Estimation of the probability of a given genetic profile requires previous knowledge of allele frequency distributions, linkage equilibrium and no departure from Hardy-Weinberg equilibrium of the genetic markers used in forensic cases. These data are usually obtained by genotyping a representative sample of individuals from the reference population of interest. However, due to different demographic histories, the genetic make up of the populations can vary significantly, even between neighboring populations. Thus, frequency estimates obtained for a given population may not fairly represent those of another population, even when they may be considered geographically or historically closely related, or part of a bigger population. One would expect that the probability of observing a specific profile can be more precisely estimated using the frequency distribution of the population to whom it belongs –i.e. ideally represented in the reference population– than using a frequency database from another population. However, it can then be easily figured out that collecting the relevant information for each existing population worldwide would be unfeasible. Besides, even if local allele frequency databases can be built or already exist, the issue of population stratification is sometimes obviated by the forensic geneticist. The simple approach of using a single (onwards referred as global) database of a country or region is very often considered.

Population substructure has been subject to extensive debate in forensic genetics for many years (e.g. [2]–[5]). In practice this issue is often ignored by a number of forensic geneticists and some authors have in part considered it to be a minor issue [6] under the assumption that human populations are not strongly stratified. Different statistical models have been developed and scientists have proposed several practical approaches to address this issue (e.g. [3], [7]–[11]). While in criminal cases, application of these methods to correct for the subpopulation effect are thought to be conservative in weighing the evidence– i.e. favoring the defendant– this has not been properly evaluated. Furthermore, the concept of “conservativeness”, although widely accepted, might be also arguable: it could be unreasonable to understate the weight of the genetic evidence when there is no need to do so. Moreover, the term “conservativeness” is also problematic in civil (e.g. paternity) cases; it may not be reasonable to give one of the parts the advantage of the uncertainty. This practice could also have a dramatic impact in incomplete paternity cases, or when only partial profiles can be typed [12]. Finally, the risk of erroneous conclusions in DNA-testing for immigration cases is also connected with this issue and has been already discussed [13].

Most recommendations to overcome these problems are based on the use of Wright's *Fst* – or *θ* –.The *Fst* was first described by Wright [14] to estimate the level of inbreeding in a population. Several statistics have been used to describe the partitioning of genetic diversity within and among populations. Wright showed that the amount of genetic differentiation between populations could also be measured using *Fst* [14], [15]. Since them, *Fst* and related statistics are among the most widely used descriptive statistics in population and evolutionary genetics [16].

Corrections by means of *Fst* have been broadly employed by forensic laboratories and recommended by the International Society for Forensic Genetics [1]. Balding and Nichols [8] introduced a formula to calculate the matching probabilities in forensic genetics incorporating *θ*. Some formulas have been provided in the literature to correct Paternity Index (*PI*) values using *Fst* (e.g. [17]), and Balding et al. [18] presented estimates of *Fst* based on data from UK and other European populations. Nevertheless, details about its routine use in forensics are scarce and somehow vague. This can be problematic because forensic laboratories may adopt these principles without solid foundation. In fact, the estimation of *Fst* is itself affected by some factors that are not always taken into account, e.g. the relative sizes of the population samples [19]. The effect of sample size on the estimation of the genetic variation in the population has also been addressed by other authors (e.g. [20]). Another problem is that it is usually assumed that all subpopulations considered in the global population share a common *θ* value, and that the value is the same across all loci. Similar concerns have been previously considered by Marchini et al. [21] and also addressed by Xu et al. [22] using a simulation approach applied to single nucleotide polymorphisms (SNP). The recent review by Meirmans and Hedrick [23] provides detailed analyses of the problems of using *Fst* and related measures to assess population structure. However, all these studies were focused on genetic association studies rather than forensic applications.

On the other hand, computation of correct values of *PI* may be important in a number of paternity cases. In several countries high values of the probability of paternity *W*, defined as *W* = *PI*/(1+*PI*), say above 0.999, are required in immigration cases and then even minor differences matters. Essen-Möller suggested 0.9973 [24].

In Argentina, some level of population substructure across the country has been observed through the analyses of commonly used forensic STR (Short Tandem Repeat) markers [25], [26]. Nevertheless, interpretation of results remains controversial. In previous articles [27], [28] we have demonstrated the impact of population substructure in the statistical interpretation of paternity testing in Argentina by analyzing its effect on the *LR* estimates in trios and duos cases. However, we did not address the ability of *Fst* (or any other statistic measure) to correct for population structure in forensic genetics.

The main goal of the present study was to quantify the real effect of *Fst* corrections [16] on *PI* values in Argentinean populations and evaluate to what extent the ‘*corrected*’ *PI* values coincide with those obtained when using the local reference database. In addition, we also aimed to evaluate the consequences of using different sampling schemes for the estimation of *Fst* values. To the best of our knowledge this is the first time where the effect of *Fst* in forensic genetics is considered by way of simulating different scenarios that use data from real population samples. These simulations allow therefore the estimation of the impact of using *Fst* in real forensics.

## Materials and Methods

### Population samples and genotyping data

A total of 1,906 genetic profiles for the 15 Short Tandem Repeats (STRs) included in the Powerplex® 16 System kit (Promega, Madison, WI) were used in this study, namely D3S1358, HUMTH01, D21S11, D18S51, PENTA E, D5S818, D13S317, D7S820, D16S539, CSF1PO, PENTA D, HUMvWA, D8S1179, HUMTPOX and FGA. More information about the data is provided in Toscanini et al. [28].

Six urban populations and two Native American populations were sampled. The geographical sources of the profiles and sample sizes are indicated in **Table 1**, as well as their respective official census population size [29]–[31]. For most of the analyses performed below we grouped the samples as urban (Buenos Aires, Neuquén, La Pampa, San Luis, Santa Cruz, Tucumán) and Native American (Toba and Colla).

**Table 1:** Census of the Argentinean population in the provinces and population groups used in the present study.

Population	Type	Sample size	*Census* population size	%	Ref.
Buenos Aires	Urban	879	15,653,341	0.01	[29], [30]
Neuquen	Urban	355	474,155	0.07	[29]
La Pampa	Urban	232	299,294	0.08	[29]
Santa Cruz	Urban	132	196,958	0.07	[29]
Tucumán	Urban	75	1,338,523	0.01	[29]
San Luis	Urban	61	367,933	0.02	[29]
Colla	Native American	43	53,106	0.08	[31]
Toba	Native American	129	47,591	0.27	[31]

Buenos Aires includes Buenos Aires city and Buenos Aires province. In the most right column, the percentage of sample size relative to the census population size is shown.

### Influence of sampling procedure on the estimation of *Fst*

A simulation-based experiment was designed to measure the influence of sampling on the estimation of *Fst* values. For this experiment, we just considered two main population groups, i.e. urban and Native Americans (see above). First, we built a sub-sample from each group by randomly retrieving 86 genetic profiles; this sampling was carried out ten times (which would allow accounting for sampling variability). Second, we computed *Fst* for every pair of sub-samples. Third, new sub-samples were obtained and *Fst* estimations were computed but this time increasing the size of the urban sub-samples by a factor of 10% in consecutive steps (to a maximum sample size of 1719) and keeping the number of profiles in the Native American sub-samples constant (*N* = 86). Han et al. [19] have employed a similar approach to evaluate the effect of unbalanced sample size in genome-wide population differentiation studies.

### Computation of *PI* from trios

The standard trio pedigrees used in Toscanini et al. [28] were considered in this study. Briefly, for each of the 1,906 real profiles in the database, a set of new profiles was created by a computer –assisted procedure routine. First, allele frequencies were obtained for all the original datasets. Second, compatible profiles for both parents of each individual were built as follows: each of the two alleles was randomly assigned to each parent; then, the other allele of each parent was randomly taken from a vector of allele population frequencies of each STR locus.

Three different panels of allele frequencies were built for *PIs* calculation: (a) the reference (local) database; (b) a global database of urban profiles; and (c) a global database of urban plus Native American profiles. Additionally, *Fst* values were estimated for the latter two databases (urban and urban+native). Next, *PIs* were calculated for each trio considering the databases described in (a), (b) and (c), and also correcting the *PI* values using the *Fst* estimates obtained for databases (b) and (c) and using the formulas reviewed by Evett and Weir (p. 179) [17].

### Statistical analyses

The main aim of the statistical analysis was to evaluate the differences between *PI* values obtained in the scenarios described above. In total, we had five different sets of *PI* values: (i) *PI*s using the reference databases in each case (Buenos Aires, Tucumán, etc.), (ii) *PI*s using urban allele frequencies (then representing a global national urban database), (iii) *PI*s using urban plus Native American allele frequencies (then representing a global national database), (iv) *PI*s considering urban allele frequencies and its corresponding *Fst* value, and (v) *PIs* considering urban plus Native American allele frequencies and the corresponding *Fst* value.

Statistical analyses were carried out as described in Toscanini et al. [28] with the necessary modifications. Thus, for each individual (*N* = 1,906), five sets of 50 *PI* values were obtained from the five scenarios described above. Several goodness-of-fit tests were employed in order to examine if each set of 50 *PI* values fit with normality (see Toscanini et al. [28] for more details); as expected the normality assumption was rejected in most of the cases. All the *PI* values were converted into natural logarithms and the normality was checked again using the same goodness-of-fit tests. The normality assumption (required to properly carry out the statistical tests below) could then be accepted for the logarithm of the *PI* values (log*PI*).

Next, for each individual an ANOVA analysis was carried out between the five sets of 50 log*PI* values. ANOVA allowed testing significant differences between the log*PI* values obtained when using the different datasets. Due to the fact that the null hypothesis of equality between sets of expected log*PI* values was always rejected (with the only exception of a single individual out of 1,906), we next used the Tukey test in order to explore statistical differences between all pairwise comparisons involving the 1,905 remaining profiles. We did not consider to apply other tests as done in Toscanini et al. [28], because the Tukey's one yielded the most conservative estimates as shown empirically by the results in Toscanini et al. [28]; see also Montgomery 2001 [32]. Tukey's test accounts for multiple test correction between all possible pairwise comparisons (given the five sample sets used in this study). However, another source of multiple tests is the fact that these comparisons are based on 1,905 profiles. Therefore, we additionally implemented a Bonferroni's adjustment based on a nominal significance value α of 0.01.

Additionally, as done in Toscanini et al. [28], for each profile we computed the weighted mean difference (*WMD*) between pairs of populations. This index quantifies the magnitude of the differences between pairs of *PI* values: for each pair of population groups (see above) *i*, *j*,
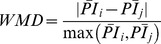
where 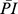 indicates the mean value for the set of 50 *PIs* obtained of each individual in each dataset. In some countries Essen-Möllers *W* (which corresponds to the posterior probability for paternity for a flat prior) is used. Since
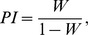

*WMD* may be expressed alternatively in terms of W as
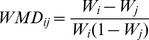
when
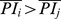
and similarly, when
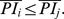



The advantage of this formulation is that it may be easier to relate to the scale of *W*, which is interpreted as a probability. A change in *W* from 0.999 (corresponding to *PI* = 999) to 0.950 (corresponding to *PI* = 19) implies a shift of two categories (from ‘Paternity practically proven’ to ‘very likely’) in Hummel's table [33] (although there appears to be no international consensus on the use of Hummel's categories in the forensic community). This change corresponds to *WMD* = 0.981.

## Results and Discussion

### Dependence of *Fst* on sampling

**Figure 1:**
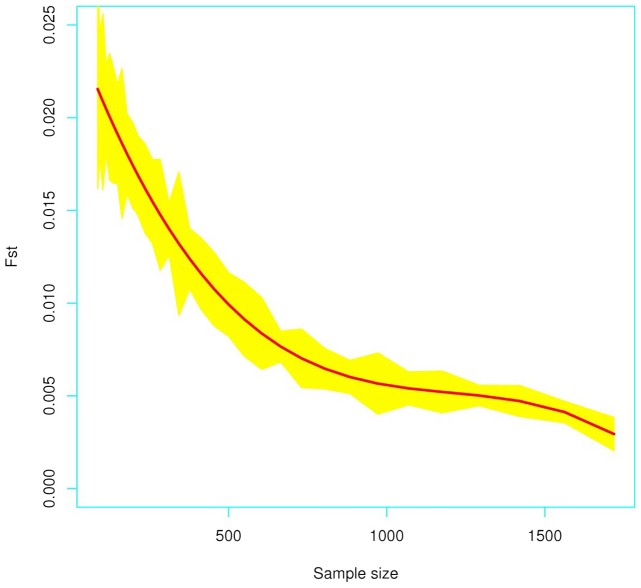
Values of *Fst* under different sampling schemes and considering original STR profiles obtained from different Argentinean population samples (Native American and Urban) as described in the text.

**Figure 1** represents the results obtained from the simulation procedure to test the influence of the sampling procedure on the *Fst* estimates. The red line in this figure shows the loess regression of the *Fst* values taking as explanatory variable the variable sample sizes, while the yellow shadow indicates sampling variability in each iteration step. As expected, the *Fst* values decrease as the urban sample size increase with an apparent trend to 0. This is obviously due to the fact that the proportion of Native Americans is progressively diluted as more urban samples are added to the computation of the *Fst*. The *Fst* values vary in this experiment about one order of magnitude. This simulation provides an idea of how different sampling schemes affect the magnitude of *Fst* values in real population scenarios (see below for further discussion).

### Evaluating the ability of *Fst* to adjust *PI* values

*PI* values in trios were obtained for the different scenarios described in Material and Methods. In brief, we considered different panels of allele frequencies, the ones derived from local (reference) databases, urban and urban+Native American populations, and also, the *PI* corrected values using two different *Fst* values, 0.00167 (urban samples) and 0.01022 (urban plus Native American samples).

Intuitively, one could assume that the local database is the ideal reference database to compute *PI* values. Therefore, the *PI* values computed using local databases can be considered to be the gold standard that best approximates the expected *PI* values.

The results indicate that there exist important differences between *PI* values computed using the proper local reference databases and computed using other database scenarios: (i) global allele frequencies (urban or urban+Native Americans), and (ii) global allele frequencies coupled with *Fst* corrections (**Table 2**). For instance, the most favorable scenario in comparison to the reference database (that is, *PI* values computed using the reference database *versus PI* values using global urban allele frequencies with *Fst* corrections), indicates that about ∼55% of the times, the difference between *PI* values is statistically significant according to the Tukey test and using Bonferroni's corrections (**Table 2**).

**Table 2:** Differences between population groups.

	U	U+N	U(*Fst*)	U+N(*Fst*)
Local *vs*. …	72.2/59.5	73.8/60.7	68.6/54.7	76.7/64.5
Local *vs.* …	22.8	23.1	20.6	22.1

Values in the first row indicate the percentages of individuals that show significant differences in pairwise comparisons under the test of Tukey for trios (the first term is for α = 0.01, while the second term is for the Bonferroni's correction assuming 1,906 comparisons). Values in the second row show the percentages of cases where WMD values were above 0.8. ‘Local’ = indicates the local (reference) database; U = urban; U+N = urban plus Native American, U(*Fst*) = urban with *Fst* corrections, U+N(*Fst*) = urban plus Native American with *Fst* corrections.

The results also indicate that in ∼21% of the cases, the *WMD* values are above 0.8; in other words, ∼21% of the times the difference in *PI* values is higher than 80% of the maximum *PI* values.

### Rationale of population sampling and computation of *Fst*

*Fst* is commonly used as a measure of population structure. Its computation entails a previous knowledge about the sub-populations to be considered and their sample sizes. Ideally, sampling should fairly represent the general population under study. However, the selection of the sub-populations that should be sampled could involve practical difficulties and/or theoretical dilemmas. For instance, in a country like Argentina, there are several Native American populations; some of them are geographically isolated from urban regions, while others are admixed to different extent with other populations of recent e.g. European ancestry. Therefore, there are populations that still remain unsampled just due to logistic difficulties for sampling collection. Moreover, the decision about the proportion of individuals that should be sampled in each region can be also problematic. A criterion to solve the latter issue could be to collect samples in a proportion similar to the official *census* of these populations. This potential solution however would lead to sampling and genotyping efforts that are unrealistic in common population genetic studies. For instance, if we consider a minimum sample size of 43 Colla individuals (as carried out in the present study), this represents 0.08% of its official population census (**Table 1**); the same proportion applied to Buenos Aires would require to sample and genotype >12,500 individuals. Representing 0.08% of the populations targeted in the present study would therefore require to genotype at least >14,000 individuals.

### Final remarks

In this study, the *Fst* values computed considering only the urban samples was 0.00167, while the addition of the Native American profiles lead to an increase of this value to 0.01022. A sampling scheme considering an equal number of the Native American populations and urban ones would certainly lead to an increase of the *Fst* values. However, it is not possible to speculate about the values taken by *Fst* under different sampling schemes because the *Fst* values can only be measured empirically. Furthermore, for a given set of sub-populations, one value of *Fst* is generally assumed but estimates could be different for distinct loci [22].

**Figure 2:**
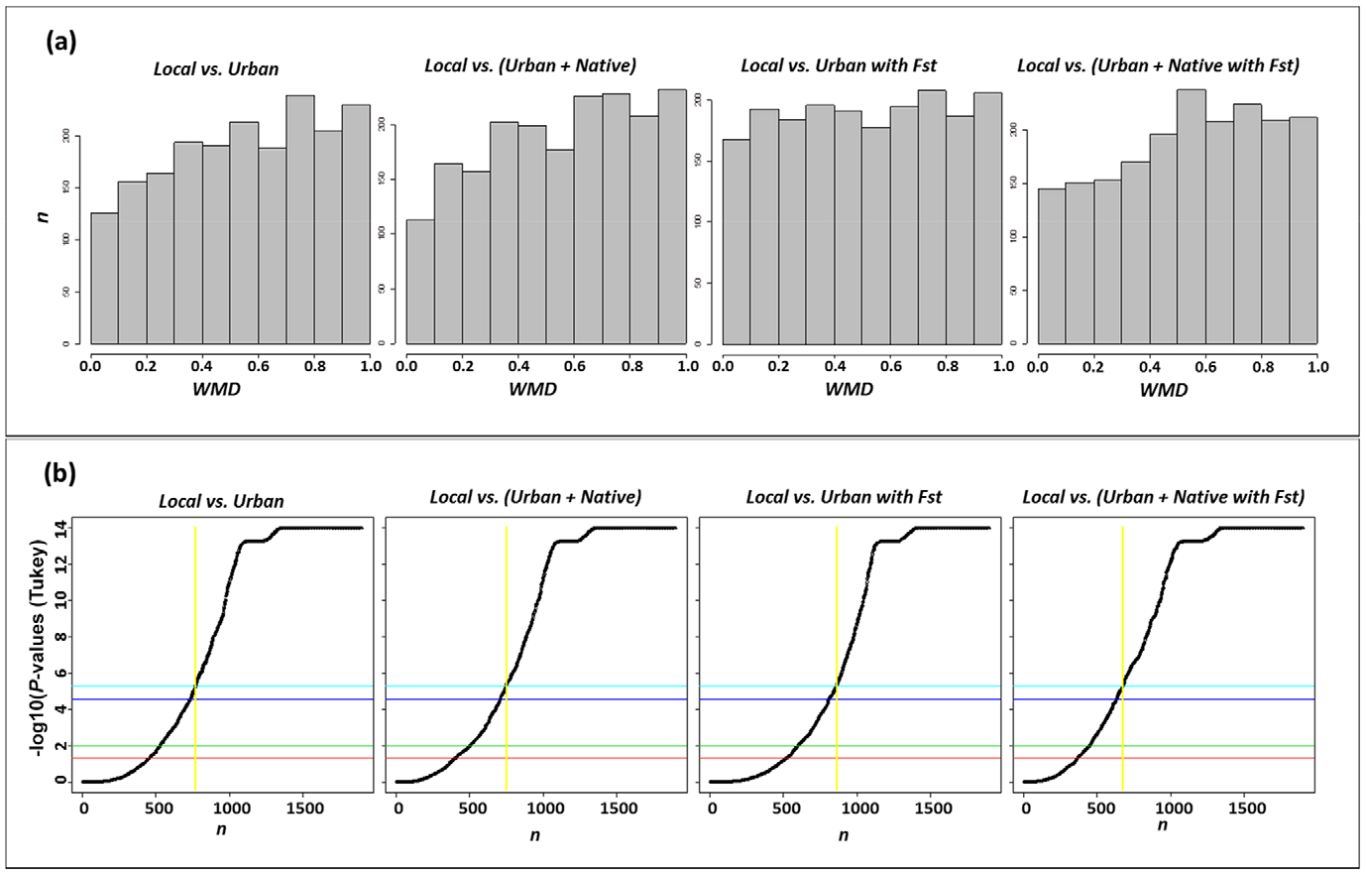
*WMD* and Tukey test *P*-values distributions for the 1,906 profiles obtained for the comparison between the local reference database and the four remaining scenarios considered. (**a**) Each histogram represents the impact on *WMD* for a given pair of frequency datasets over the 1,906. (**b**) the curve represents the minus log10(*P*-values) (Tukey test) obtained for the difference between the *PI* values for each case. The horizontal lines represent from bottom to top the log10 values for α = 0.05, α = 0.01, and the respective values for Bonferroni corrections. Cases on the right hand side of the vertical yellow line correspond to the cases where the differences where statistically significant for α = 0.01 after Bonferroni correction.

The procedure recommended by the general forensic community to deal with population stratification is the computation of *PI* using a panel of global allele frequencies (e.g. urban or urban+Native American populations) coupled with a correction based on the ‘appropriate’ *Fst* value. Note however that, in general, *Fst* corrections do not have an important impact on the *PI* values computed using the corresponding pooled database, as can be seen by comparing the values of columns 1 and 2, and the values in columns 3 and 4 in the first row of **Table 2**. In addition, as displayed in the histograms of **Figure 2(a)**, although there is an increase in the number of cases with lower *WMD* values when applying the *Fst* correction, the number of statistically significant differences between the *PI*s obtained with the local reference database and the ones obtained using the other databases considered are still very important (values on the right side of the vertical yellow line in each panel of **Figure 2(b)**). In other words, *F_ST_* corrections do not properly approximate the results obtained under the ideal scenario represented by the local reference database.

It is also noteworthy that applying the *Fst* correction, *PI* values are not always lower than the ones obtained using the proper reference database (**Table 3**). Columns two, four and six in this **Table 3** indicate that there is a remarkable number of *PI*s that are one, two or three orders of magnitude greater using the *Fst* correction than the reference values (i.e. using the local database), which is similar to the number of *Fst*-corrected *PI* values that are lower than the reference ones. This reflects that the assumption that the unknown subpopulations are being fairly represented by the global population database might not be true, and that the use of *Fst* for this purpose is not always conservative.

**Table 3:** Numbers of pairwise comparisons exhibiting a difference of a given order of magnitude using *Fst* corrections *versus* the local database.

	[1]	[2]	[3]	[4]	[5]	[6]
Urban	2 (0.1)	103 (5.9)	0 (0.0)	1 (0.1)	0 (0.0)	0 (0.0)
Native American	70 (40.7)	5 (2.9)	26 (15.1)	2 (1.2)	3 (1.7)	0 (0.0)
**Total**	**72 (3.8)**	**108 (5.7)**	**26 (1.4)**	**3 (0.2)**	**3 (0.2)**	**0 (0.0)**

For the computation, the Urban+Native American frequency database with the corresponding *Fst* corrections was employed. The values indicate the number of Urban or Native trios that exhibited a *PI* value higher than 1, 2 and 3 orders of magnitude (Columns 2, 4 & 6) and lower than 1, 2 and 3 orders of magnitude (Columns 1, 3 & 5) using the Urban+Native American database with the corresponding *Fst* corrections relative to the *PI* values obtained with the reference database. [1]: *PI(Local)*(×100)>*PI(Fst)*>*PI(Local)*(×10); [2]: *PI(Local)*(×0.01)<*PI(Fst)*<*PI(Local)* (×0.1); [3]: *PI(Local)*(×1000)>*PI(Fst)*>*PI(Local)*(×100); [4]: *PI(Local)*(×0.001)<*PI(Fst)*<*PI(Local)* (×0.01); [5]: *PI(Fst)*>*PI(Local)* (×1000); [6]: *PI(Fst)*<*PI(Local)* (×0.001). ‘*PI(Local)*’ indicates the *PI* for the local (reference) database. ‘*PI(Fst)*’ indicates the *Fst*-corrected *PI* for the Urban+Native American database. In brackets are the corresponding percentages.

One could also argue that the *Fst* values are usually “low” for most human populations, and even that the differences between the reference *PI* values and the *Fst*-corrected ones might not be relevant for decisions in court. This could however give rise to some thorny questions: what is a “low” *Fst* value?; or when is the decision process between paternity/non-paternity compromised? There may be no simple answer for most of these questions.

In spite of their magnitudes, differences do exist when applying *Fst* correction, thus extending the issue beyond any academic discussion since there is a real impact on routine casework.

The present study has attempted to evaluate the suitability of *Fst* corrections to deal with populations sub-structure in the computation of *PI* values in paternity trio cases, using for the first time a simulations based on real datasets and therefore mirroring cases that could be occurring in real casework. The results indicate that:

- there is not an obvious and objective way to measure real *Fst* values from a given population since the computation of *Fst* is strongly dependent on sampling strategy. Furthermore, we noticed that low *Fst* values (the range evaluated in the present study was 0.00167 to 0.0102) coupled with the way these *Fst* values are implemented in the computation of *PI*, can significantly influence the final *PI* values; in Europe, *Fst* values are probably not significantly lower than 0.0102 and, as already advanced in 1996 by Balding et al.: “values of *Fst* appropriate to forensic applications in Europe are too large to be ignored” [18];
- the common practice in forensic paternity cases of using global databases even when these *PI* values are corrected using *Fst*, might be inappropriate in a number of cases,
- global databases might not properly represent the genetic characteristics of any subpopulation, and
- the, sometimes lightly accepted, thought that the use of *Fst* is conservative does not always hold.

In summary, the results indicate that the role of local reference databases cannot easily be substituted by other sample schemes and methods to correct for stratification. When possible, the population of interest should be properly sampled in order to represent as much as possible of its genetic heterogeneity.
